# Editorial: Saturated fat: metabolism, nutrition, and health impact

**DOI:** 10.3389/fnut.2023.1208047

**Published:** 2023-06-14

**Authors:** Fabian M. Dayrit

**Affiliations:** Department of Chemistry, Ateneo de Manila University Loyola Heights, Quezon City, Philippines

**Keywords:** saturated fatty acid, medium chain fatty acid, long chain fatty acid, medium chain triglyceride, lipid metabolism

Saturated fatty acids are simple linear chains of singly-bonded carbon atoms which are differentiated only by chain length. However, the simplicity of their chemical structure belies the complexity of their metabolic properties and their nutritional and health impacts. Linear saturated fatty acids are generally divided into the following groups: short-chain (SCFA, C2 to C4), medium-chain (MCFA, C6:0 to C12:0), and long-chain (LCFA, C14:0 to C18:0). MCFAs are naturally found in a limited number of sources, such as mammalian milk and seed oils, in particular coconut oil.

The objective of this collection of research and review papers is to highlight the unique roles that various saturated fatty acids play. These articles show that, despite their structural simplicity and apparent similarities, various saturated fatty acids, in particular MCFAs, have different metabolic properties which impact on nutrition and health. This collection of research articles on saturated fatty acids covers topics ranging from nutrition of the pregnant mother and infant to diet, cancer, obesity, diabetes and other metabolic diseases.

Although the fatty acid composition of fats and oils are the usual focus of interest, the position of the fatty acid on the triglyceride can affect their digestion and absorption. Yuan et al. showed how the digestion and absorption of different MCFAs in infants are influenced by the position of the fatty acid on the MCT. Human breastmilk, which is made up of about 15% MCFA, is an important source of infant nutrition and MCFAs play a role in the development of the immune system. The authors note how MCFAs modulate the infant's gut microbiota which can prevent obesity and enhance brain development. There is growing interest in the triglyceride structure of MCFAs for the development of healthier formula milk for infants. This knowledge can also be used in the formulation of nutritional products for geriatric care as well.

Sun et al. studied gestational diabetes mellitus (GDM) in pregnant women analyzing fatty acids in the blood plasma, from myristic acid (C14:0) to lignoceric acid (C24:0). They found that high concentrations of palmitic acid (C16:0) coupled with low concentrations of very long chain saturated fatty acids (C20:0 to C24:0) were positively associated with GDM. This study provides more information on the observed positive correlation of palmitic acid and inverse correlation of very long chain saturated fatty acids on hepatic insulin resistance through their ceramide derivatives. This highlights the impact of the diet of pregnant women and the importance of studying lipid metabolism in greater depth.

Nonaka et al. studied the effects in mice of MCFAs on glucose homeostasis and lipid metabolism through gut hormone GLP-1. Their mouse study showed that tridecanoate (the MCT of C10:0) improved glucose homeostasis and prevented obesity induced by a high fat (lard) diet. This highlights the beneficial properties of MCFAs and shows that saturated fatty acids of different chain lengths have different effects. This study showed that MCFAs are not only energy-rich compounds but are also able to influence metabolic processes. These results may contribute to the development of MCTs as functional foods for the prevention of metabolic disorders, such as obesity and type 2 diabetes.

The paper of Watanabe and Tsujino contributed to the theme of the applications of MCTs in functional foods. They recalled that MCTs were used in the 1960's mainly as a source of energy. However, recent discoveries have shown their potential in treating many metabolic dysfunctions that afflict modern society. MCTs have been shown to promote protein anabolism and inhibit catabolism which can delay frailty in the elderly. Of particular interest today is the relatively large ingestion of MCTs to elevate ketone bodies which can be used to support nutrition in conditions such as cancer, Alzheimer's disease, and epilepsy. Recent research is providing more knowledge for the wider use of MCTs in food.

All of the papers in this collection call for more studies on saturated fatty acids, in particular MCFAs, so that their full potential can be developed. The new knowledge on saturated fatty acids, which are the simplest biomolecules of life, are leading to a new paradigm regarding their health benefits. With the rise of obesity worldwide, scientifically-informed guidelines on the use of MCFAs is urgently needed.

## Author contributions

The author confirms being the sole contributor of this work and has approved it for publication.

